# Intra-observer, inter-experiment and inter-observer variability of left ventricular volumes and mass measurements in mice using an 11.7 Tesla MRI

**DOI:** 10.1186/1532-429X-15-S1-P282

**Published:** 2013-01-30

**Authors:** Laetitia Vanhoutte, Olivier Feron, Jean-Luc Balligand, Bernard Gallez, Hrag Esfahani, Stéphane Moniotte

**Affiliations:** 1 FATH, UCL, Brussels, Belgium; 2 Pediatrics, UCL, Brussels, Belgium; 3 REMA, UCL, Brussels, Belgium

## Background

In cardiovascular research, there is an increasing interest in high-field MRI to characterize cardiac anatomy and function in small rodents. However, data on reproducibility of the method are scarce. Our aim was to evaluate the intra-observer, inter-experiment and inter-observer variability of left ventricular (LV) mass and volumes measurements in mice using an 11.7T MRI scanner.

## Methods

Seven 10 weeks-old C57Bl6J male mice were studied, including 4 animals with a surgical transverse aortic constriction in order to produce cardiac hypertrophy. 3 were sham (control) mice.

During the entire procedure, animals were anaesthetized with Isoflurane 1-3%, in a temperature-controlled setting. Scan were retrospectively gated for electrocardiogram (ECG) and respiration. Imaging was performed on a 11.7 Tesla Bruker MR scanner. Cardiac scout images were obtained in the conventional planes with a tripilot sequence. A 2D cine FLASH sequence was applied to acquire a set of 7 contiguous short axis images covering the entire ventricles, perpendicular to the LV long axis. One day after the procedure, mice were scanned again under the same conditions.

The LV systolic function was assessed from each stack of images by tracing epicardial and endocardial borders, including papillary muscles, on the Segment software (Medviso, Sweden). End-diastolic (EDV), end-systolic (ESV) and stroke volume (SV) were determined (µl). LV ejection fraction (EF, in %) and LV mass (mg) were subsequently deduced. One stack of images was analyzed twice by the same person, and once by an independent observer.

## Results

Accurate quantitative data were obtained in all animals. The Table 1 lists the mean ± SD values and the Bland-Altman and Pearson analysis results. The best reproducibility was obtained for the measurements of LV mass and EDV, with a coefficient of variability (Cov) between 1.70 and 12%. More variation was found in ESV measurement (CoV 1-70-22.40%). Results for SV and EF were the less reproducible, with a CoV between 14.52 and 31.45%. The Pearson correlation coefficient indicates good correlation between all methods for all parameters (r square between 0.81 and 0.99), except for the SV, particularly in terms of inter-experiment reproducibility (r square =0.03).

**Table 1:** Intra-observer, inter-experiment and inter-observer variability analysis.

Intraobserver variability	LV mass	EDV	ESV	SV	EF
Mean ± SD data 1	93,00 ± 8,018	76,57 ± 6,629	42,14 ± 8,606	34,29 ± 2,792	47,86 ± 6,674

Mean ± SD data 2	93,71 ± 7,643	76,86 ± 6,857	40,00 ± 10,04	37,14 ± 3,744	52,43 ± 8,569

Bias	-0,7143	-0,2857	2,143	-2,8571	-4,5714

Limits of agreement	-12,62 to 11,19	-3,990 to 3,418	-11,55 to 15,84	-15,43 to 9,715	-23,23 to 14,09

SDD	6,07	1,89	6,99	6,41	9,52

Coefficient of variability	6,51%	2,46%	1,70%	17,96%	18,98%

r square	0,92	0,99	0,92	0,58	0,84

Interexperiment variability	LV mass	EDV	ESV	SV	EF

Mean ± SD data 1	93,00 ± 8,018	76,57 ± 6,629	42,14 ± 8,606	34,29 ± 2,792	47,86 ± 6,674

Mean ± SD data 3	96,43 ± 7,970	73,14 ± 7,557	41,29 ± 9,526	31,71 ± 3,834	47,14 ± 7,658

Bias	-3,429	3,429	0,8571	2,571	0,7143

Limits of agreement	-20,70 to 13,84	-13,46 to 20,32	-13,25 to 14,96	-8,450 to 13,59	-12,80 to 14,23

SDD	8,81	8,619	7,198	5,623	6,897

Coefficient of variability	9,64%	12%	17,25%	17,04%	14,52%

r square	0,83	0,81	0,92	0,71	0,89

Interobserver variability	LV mass	EDV	ESV	SV	EF

Mean ± SD data 2	93,71 ± 7,643	76,86 ± 6,857	40,00 ± 10,04	37,14 ± 3,744	52,43 ± 8,569

Mean ± SD data 4	95,14 ± 8,379	67,57 ± 7,593	34,29 ± 7,296	33,57 ± 1,360	52,71 ± 5,384

Bias	-1,43	9,29	5,71	3,57	-0,29

Limits of agreement	-12,57 to 9,71	-1,43 to 20,00	-10,59 to 22,02	-18,22 to 25,36	-22,16 to 21,59

SDD	5,48	5,47	8,32	11,12	11,16

Coefficient of variability	6,02%	8%	22,40%	31,45%	21,23%

r square	0,94	0,93	0,97	0,03	0,84

## Conclusions

In our hands, reproducibility was excellent for LV mass and EDV, good for ESV and EF to a lesser extent, and more limited for the stroke volume, with a bad inter-observer correlation. This highlights the need to interpret our results by taking into account these limitations, focusing on more reproducible parameters when interpreting data.

## Funding

This work was supported by grants from the Fonds de la Recherche Scientifique FRS-FNRS.

